# Meta-topolin stimulates de novo shoot organogenesis and plant regeneration in cassava

**DOI:** 10.1007/s11240-017-1315-3

**Published:** 2017-10-04

**Authors:** Raj Deepika Chauhan, Nigel James Taylor

**Affiliations:** 1 Donald Danforth Plant Science Center, 975 North Warson Road, St. Louis, MO 63132, USA

**Keywords:** Cassava, Meta-topolin, Organogenesis Shoot regeneration

## Abstract

A novel protocol for de novo shoot organogenesis from cassava has been developed utilizing meta-topolin to stimulate shoot regeneration from leaf, petiole and stem internode explants. While use of meta-topolin alone was capable of inducing shoot regeneration, a two-stage system combining meta-topolin with 2,4-D in a first stage medium, followed by subculture onto elevated levels of meta-topolin, was superior for inducing multiple shoot regeneration events in more than 35% of explants in cultivar TME 7. Caulogenesis was achieved in eleven additional cultivars. Metatopolin was also found to be beneficial for stimulating shoot regeneration from somatic embryos and cotyledon explants. The shoot organogenesis techniques described enhance the capacity of existing embryogenic systems and present previously unavailable morphogenic pathways for developing genetic transformation and gene editing technologies in cassava.

Reliable and efficient methods for de novo plant regeneration are central to the application of genetic transformation and genome editing technologies. In the tropical root crop cassava (*Manihot esculenta*), somatic embryogenesis is the morphogenic process most frequently employed for the regeneration of transgenic plants (Chavarriaga-Aguirre et al. 2016; Liu et al. 2011). Although effective, these totipotent tissue systems require multi-step subculture procedures that are resource-intensive and time-consuming. Development of alternative regeneration pathways for this crop therefore remains important.

Plant regeneration through indirect organogenesis is exploited in basic and applied research across many plant species (Xu and Huang 2014). Descriptions of organogenesis in cassava, however, remain limited to adventitious shoot regeneration from the cotyledons of somatic embryos (Li et al. 1998), and single reports from leaf (Musio et al. 1998) and internode explants (Tilquin 1979). We have developed a novel and reproducible protocol for organogenesis based on the use of meta-topolin (*m*T), a naturally occurring aromatic cytokinin originally isolated from leaves of *Populus x robusta* (Horgan et al. 1975). When employed in cassava, *m*T was found to stimulate shoot regeneration from a range of explant types in a manner not previously reported in this species.

Various types and concentrations of cytokinins were tested for their ability to induce shoot organogenesis from leaf explants. Leaf lobe explants (4–6 mm in length) were excised from 6- to 8-week- old in vitro grown mother plantlets of cassava cultivars TME 7 and TME 204 (Chauhan et al. 2015) and placed on Murashige and Skoog’s (Murashige and Skoog 1962) basal media containing 2% w/v sucrose (MS2), solidified with 0.8% Noble agar and supplemented with varying concentrations of kinetin, 6-benzylaminopurine (BAP), zeatin or thidiazuron (TDZ). Two tissue types developed from the explants, identified as pale yellow, non-organogenic callus or compact, green nodular tissue (Fig. 1c, d). Shoot regeneration was observed from 11 and 5% of the explants of TME 204 and TME 7, respectively over a period of 5–10 weeks on media containing BAP and zeatin, respectively (data not shown). In all cases, shoots regenerated from the compact, green nodular tissues. In an effort to improve the caulogenic response, the effect of *m*T was assessed on different explant types. Leaf lobe, leaf-petiole [whole immature leaf with 1.0-1.5 mm of the petiole attached (Fig. 1a)], petiole and stem internode explants were excised from in vitro grown TME 7 mother plants that had received a four-week pre-treatment on MS2 medium supplemented with 2 μM *m*T. Explants were placed on MS2 medium supplemented with 6 μM *m*T followed by subculture onto fresh medium of the same type every 3 weeks. Compact green nodular tissues were produced from all explant types with a significantly higher response observed from leaf lobes at 72% and leaf-petioles at 56% (*p* < *0.05*), compared to the stem internode and petiole explants. While all green nodular tissues produced non-regenerating foliose structures from their surface (Fig. 1), only 2% of leaf lobe-derived explants subsequently underwent shoot organogenesis. In contrast, shoot regeneration frequencies of 22, 20, and 10% were achieved from leaf-petiole, stem internode and petiole explants, respectively (Table 1).

**Fig. 1:**
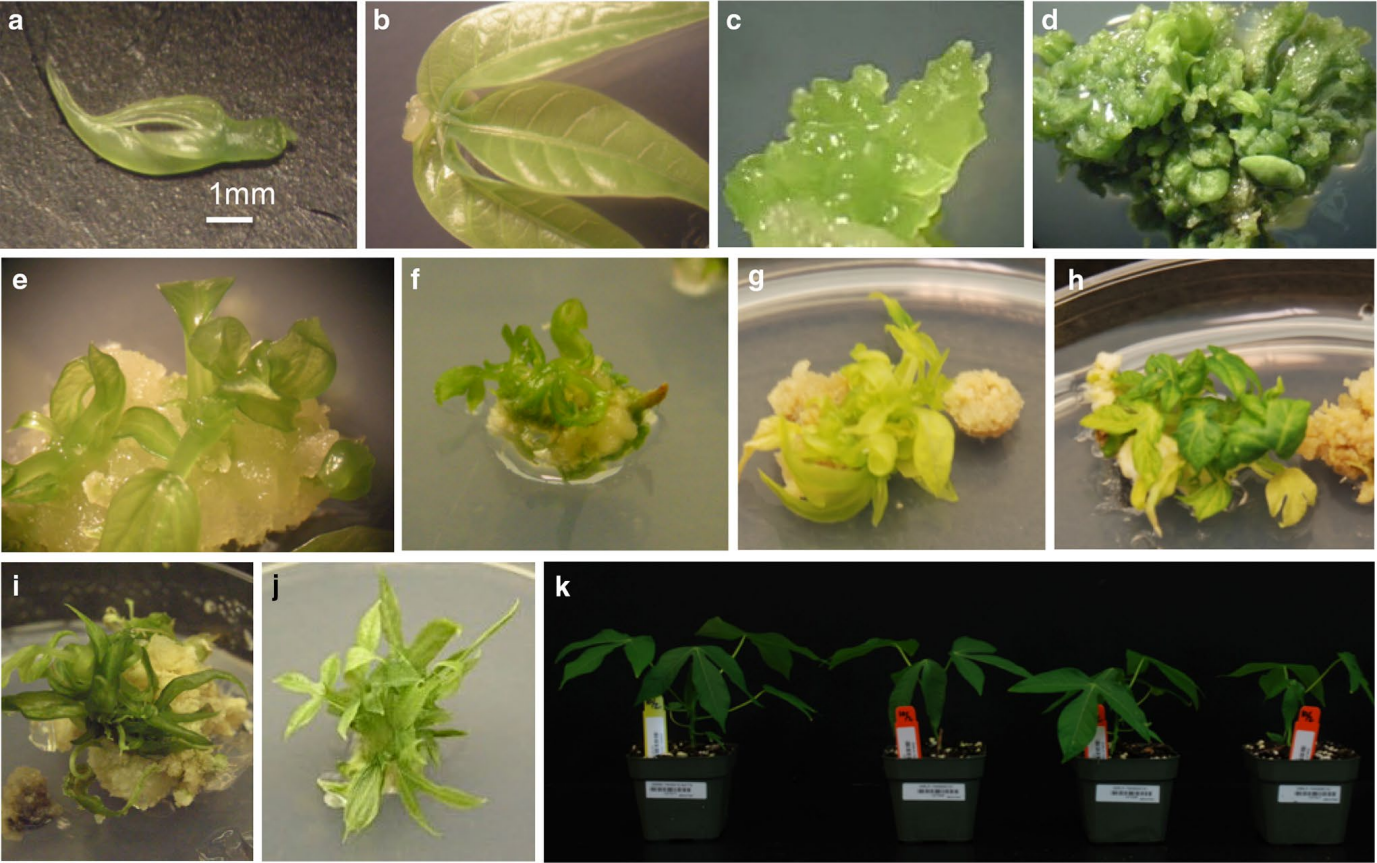
De novo shoot organogenesis from leaf and cotyledon explants of cassava. **a** Leaf-petiole explants, **b** callus induction from the wound site of leaf-petiole explant on MS2 media supplemented with 1 μM 2,4D and 1 μM *m*T (Stage 1) after 7 days culture, **c** green nodular tissues formed on MS2 media supplemented with 6 μM *m*T (Stage 2) after 14 days culture, **d** green nodular tissues with foliose structures formed on Stage 2 media after 28 days culture, **e** green bud structures and shoot regeneration from leaf-petiole explant of TME 7 after 6 weeks culture using two-stage culture method, **f** shoot regeneration from leaf-petiole explant of cv. TME 14 after 5 weeks culture using two-stage culture method, **g** shoot regeneration from leaf-petiole explant of cv. TMS 98/0002, **h** shoot regeneration from cv. 60444 from leaf-petiole explant after 5 weeks culture using two-stage culture method, **i** shoot regeneration from cotyledon explants of cv. TME 204, **j** proliferation of shoots on MS2 medium, **k** robust plants of cultivar TME 7 obtained regenerated leaf-petiole explants established under greenhouse conditions

**Table 1:** Effect of meta-topolin on shoot regeneration from explants of cassava cultivar TME 7

Explant type	Average percent explants forming green nodular tissues	Average percent explants forming shoots	Average percent green nodular tissues regenerating shoots
Leaf lobe	72.0 ± 3.6	2.0 ± 0.9	2.2 ± 1.0
Leaf-petiole	56.0 ± 5.4	14.0 ± 3.0	22.0 ± 5.2
Petiole	6.0 ± 1.9	2.0 ± 0.9	10.0 4.5
Stem internode	12.0 ± 2.2	4.0 ± 1.8	20.0 ± 8.9

Explants were excised from in vitro shoot cultures preconditioned on MS based medium containing 2 μM *m*T for 4 weeks. Immature leaf lobes, leaf-petiole explants (whole leaf plus 1–1.5 mm petiole) and stem internodes were cultured on MS based medium containing 6 μM *m*T for 3 weeks followed by subculture on same media type. Data represents the average response ± SE. Ten explants were cultured per Petri dish and five Petri dishes established per explant type. Number of explants forming green nodular tissues and shoots were counted after 4 to 6 weeks of culture

The relatively high shoot regeneration potential of leaf -petiole explants was utilized to investigate the effect of combining the potent auxin 2,4-dichlorophenoxyacetic acid (2,4-D) with *m*T. Leaf-petiole explants were excised from mother plants of cvs. TME 7 and TME 14 pre-treated with 2 μM *m*T for 4 weeks, and cultured on MS2 medium (Stage 1) supplemented with 1, 6 or 12 μM 2,4-D and 1 or 6 μM *m*T (Fig. 2). After 7 days, the explants developed white to pale yellow colored callus at their wounded site (Fig. 1b). When subcultured onto MS2 medium devoid of auxin and containing 6 μM *m*T (Stage 2), this callus became compact, turned green in color and developed nodular structures (Fig. 1c,d). A maximum of 70% of the explants formed green compact nodular tissues when cultured on a stage 1 medium containing 1 μM 2,4-D and 1 μM *m*T. Adventitious shoots were first seen regenerating from green compact nodular tissues 4 weeks after subculture onto the Stage 2 medium supplemented with 6 μM *m*T (Fig. 1e–h). A maximum regeneration frequency of 36%, with an average of two shoots per explant, was achieved when TME 7 leaf-petioles were cultured on Stage 1 media containing 1 μM 2,4-D and 1 μM *m*T, followed by transfer to 6 μM *m*T. TME 14 was less caulogenic than TME 7 under these conditions, with 17% of the explants undergoing de novo shoot regeneration (Fig. 2). Plants regenerated through culture on *m*T were transferred to soil and established in the greenhouse following procedures described by Taylor et al. (2012). Rooted plants established at a 100% survival rate were robust and showed no abnormal phenotypes compared to controls (Fig. 1k).

**Fig. 2:**
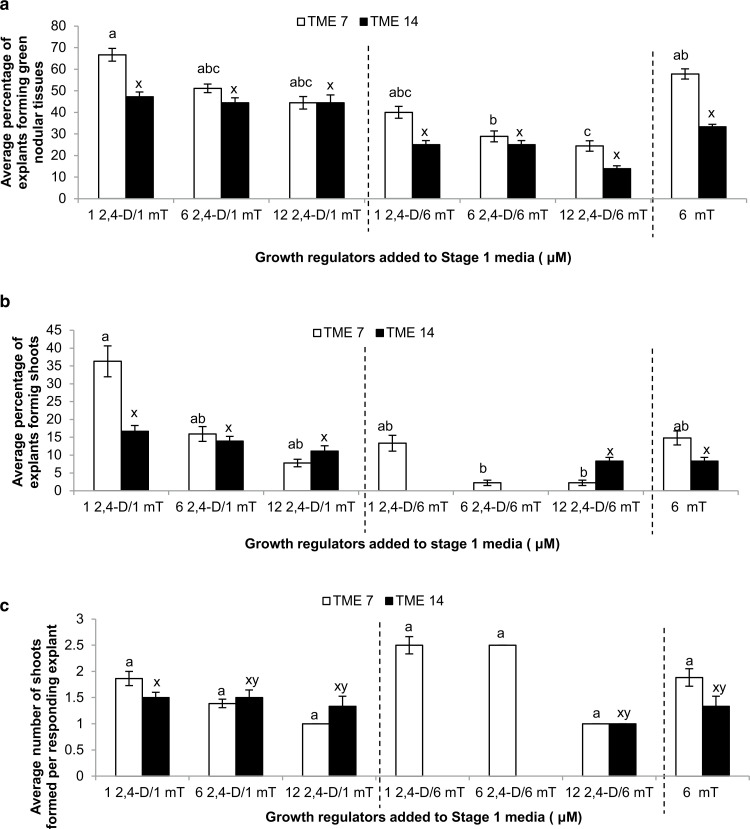
Shoot organogenesis from leaf-petiole explants of cassava cv. TME 7 and TME 14 after 8 weeks culture. **a** Percent explants forming green nodular tissues when cultured on MS media supplemented with 2,4D and *m*T (Stage 1) for 7 days followed by transfer to MS media supplemented with 6 μM *m*T for 2–3 weeks, **b** percent explants forming shoots after 5–8 weeks after explanting, **c** average number of shoots formed per responding leaf-petiole explant after 8 weeks culture on MS2 media supplemented with 6 μM *m*T. Data represented as average response ± SE. The experiment was repeated three times for TME 7 and two times for TME 14. Means that do not share a letter for each of the cultivars are significantly different at 95% confidence using Turkey pairwise comparison.

Meta-topolin has been shown to promote in vitro shoot proliferation and improve quality of shoots in several plant species (Aremu et al. 2012). Its use in cassava has here enabled the first report of reproducible induction of shoots through de novo organogenesis from somatic tissues such as the petiole and stem internode. In order to determine if the caulogenic effects of *m*T extended into a wider range of cassava cultivars, the 41 African, American and Asian cassava accessions were assessed for their response to culture on this cytokinin. Green nodular tissues were successfully induced from leaf-petiole explants of 25 additional cultivars after 3–4 weeks of culture on the two-stage method and shoots successfully regenerated from BRA 83, CR 142, GUA 86, MBRA 685, Mbundamali, M. Col 2436, Mercury, 60444, TME 7, TME 14, TME 204 and TMS 98/0002 (Fig. 1e–h).

Effective use of cytokinins is essential for germination of somatic embryos in cassava (Guohua 1998; Mongomake et al. 2015; Taylor et al. 2012), and for induction of shoot organogenesis from cotyledon explants (Li et al. 1998). The effect of *m*T on these processes was assessed and compared to the commonly used BAP (Mongomake et al. 2015; Raemakers et al. 1993). Organized embryogenic structures (OES) were induced from TME 204 and TME 7 leaf lobe explants cultured on Driver and Kuniyuki Walnut/Juglans basal salts (Driver and Kuniyuki 1984) (DKW), supplemented with MS vitamins, 2% w/v sucrose, 50 μM picloram and solidified with 0.8% Noble agar (Chauhan et al. 2015). After 4 weeks, small clumps of embryogenic tissue (3–4 mm in diameter) consisting of four to five OES units were subcultured onto MS2 media containing 2 μM *m*T and solidified with 0.22% gelzan. After 2–3 weeks, individual cotyledon stage embryos were separated and transferred to the same medium type to induce shoot germination. *m*T was found to stimulate recovery of greater numbers of mature cotyledon stage embryos and a higher frequency of plantlet germination than BAP (Table 2). Plantlet germination frequencies of 85 and 43% were achieved from cotyledon stage somatic embryos of TME 7 and TME 204 respectively, that were higher than those reported previously (Mongomake et al. 2015; Raemakers et al. 1993).

**Table 2:** Effect of meta-topolin on somatic embryo maturation and plant regeneration from organized embryogenic structures

Cultivar	TME 7		TME 204	
Growth regulator	Average number of cotyledon stage embryos formed per OES colony	% cotyledon stage embryos germinating to form plants	Average number of cotyledon stage embryos formed per OES colony	% cotyledon-stage embryos germinating to form plants
*m*T (2.0 μM)	4.2 ± 0.1	84.7 ± 1.2	2.6 ± 0.1	42.9 ± 1.4
BAP (4.4 μM)	2.6 ± 0.1	71.1 ± 1.7	2.3 ± 0.1	37.7 ± 1.5

Eight OES colonies (4–5 OES units per colony) were placed in each Petri dish and 10 Petri dishes established per treatment. OES colonies were cultured onto MS based media containing 2 μM *m*T and solidified with 0.22% gelzan and tissues subculture onto fresh media of the same type every 2 weeks. Cotyledon stage somatic embryos were separated and cultured individually on same media type to stimulate germination. Data represents average response ± SE. The average numbers of green cotyledon stage embryos formed per OES colony were recorded after 4 weeks of culture and embryo germination after 8 weeks of culture on MS based media containing 2 μM *m*T

The potential of *m*T to induce organogenesis from somatic embryo derived cotyledon explants was also assessed. Cotyledon-stage embryos of cv. TME 204 were generated on MS2 medium containing 2 μM *m*T. The shoot organogenesis was induced using the two-stage 2,4-D/*m*T culture system developed for leaf-petiole explants (Fig. 3). A shoot regeneration frequency of 66% was achieved when pale yellow, translucent cotyledon explants were pre-cultured for 7 days on MS2 media supplemented with 1 μM 2,4-D and 1 μM *m*T followed by transfer to medium containing 6 μM *m*T (Fig. 3). The organogenic potential of explants obtained from the green mature cotyledon stage was significantly lower than the pale yellow cotyledon stage embryos, confirming observations reported by Li et al. (1998). *m*T was found to stimulate shoot regeneration from cotyledon at frequencies similar to or greater than those reported previously from this explant type (Guohua 1998; Li et al. 1998).

**Fig. 3:**
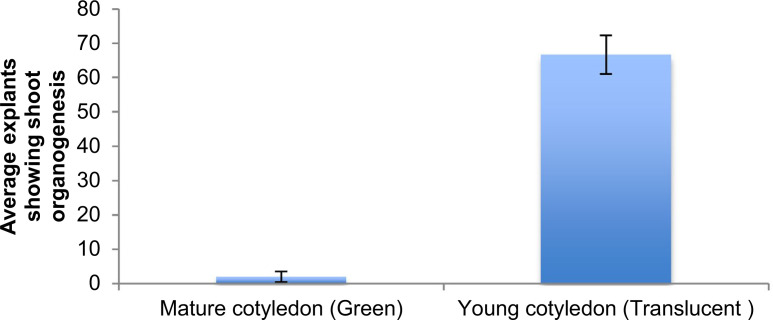
Shoot organogenesis from cotyledon explants of cassava cv. TME 204 after 8 weeks of culture on two-stage 2,4D/*m*T culture system. Data is represented as average response ± SE

To date, totipotent systems in cassava have been dependent on the use of potent auxins, such as picloram and 2,4-D, to induce embryogenic tissues from which plants can be regenerated. Explant types capable of this response have been limited to axillary meristems and immature leaves (Chauhan et al. 2015; Taylor et al. 2012). We report here that petioles, leaves and stem internode explants can be induced to undergo de novo caulogenesis when cultured on medium supplemented with the cytokinin *m*T. Use of low levels of 2,4-D increased the frequency of shoot regeneration (Fig. 2b), but de novo caulogenesis could also be achieved by culture on *m*T alone. A total of 12 cultivars underwent shoot regeneration when exposed to *m*T, indicating its potential application across cassava cultivars from diverse origins. Its effectiveness was also shown for improving plant recovery from somatic embryos and inducing shoot organogenesis from cotyledon explants. The culture processes described have yet to be fully optimized, but it is considered that use of *m*T brings important new opportunities for development of simple and rapid plant regeneration systems in cassava with potential applications in genetic transformation and gene editing for this important staple crop.

## Abbreviations

BAP: 6-Benzylaminopurine
DKW: Driver and Kuniyuki Walnut
2,4-D: 2,4-Dichlorophenoxyacetic acid
*m*T: Meta-topolin
MS: Murashige and Skoog
NAA: Naphthalene acetic acid
TDZ: Thidiazuron

## References

[cit0001] Aremu AO, Bairu MW, Doležal K, Finnie JF, Van Staden J (2012) Topolins: a panacea to plant tissue culture challenges? Plant Cell Tiss Organ Cult 108. doi:10.1007/s11240-011-0007-7

[cit0002] Chauhan RD, Beyene G, Kalyaeva M, Fauquet CM, Taylor N (2015) Improvements in Agrobacterium-mediated transformation of cassava (Manihot esculenta Crantz) for large-scale production of transgenic plants. Plant Cell Tiss Org 121:591–603. doi:10.1007/s11240-015-0729-z

[cit0003] Chavarriaga-Aguirre P, Brand A, Medina A, Prías M, Escobar R, Martinez J, Díaz P, López C, Roca W, Tohme J (2016) The potential of using biotechnology to improve cassava: a review. In Vitro Cell Dev Biol Plant 52:461–478 27818605 10.1007/s11627-016-9776-3 PMC5071364

[cit0004] Driver J, Kuniyuki A (1984) In vitro propagation of Paradox walnut rootstock. Hort Science 19:507–509

[cit0005] Guohua M (1998) Effects of cytokinins and auxins on cassava shoot organogenesis and somatic embryogenesis from somatic embryo explants. Plant Cell Tiss Org 54:1–7. doi:10.1023/A:1006065120629

[cit0006] Horgan R, Hewett EW, Horgan JM, Purse J, Wareing APF (1975) A new cytokinin from *Populus x robusta*. Phytochemistry 14:1005–1008. doi:10.1016/0031-9422(75)85176-4

[cit0007] Li H, Guo J, Huang Y, Liang C-Y, Liu H-X, Potrykus I, Puonti-Kaerlas J (1998) Regeneration of cassava plants via shoot organogenesis. Plant Cell Rep 17:410–414. doi:10.1007/s002990050416 30736581

[cit0008] Liu J, Zheng Q, Ma Q, Gadidasu KK, Zhang P (2011) Cassava genetic transformation and its application in breeding. J Integr Plant Biol 53:552–569. doi:10.1111/j.1744-7909.2011.01048.x 21564542

[cit0009] Mongomake K, Doungous O, Khatabi B, Fondong VN (2015) Somatic embryogenesis and plant regeneration of cassava (Manihot esculenta Crantz) landraces from Cameroon. Springerplus 4:477. doi:10.1186/s40064-015-1272-4 26361578 PMC4559553

[cit0010] Murashige T, Skoog F (1962) A revised medium for rapid growth and bio assays with tobacco tissue cultures. Physiol Plant 15:473–497

[cit0011] Musio I, Chaput MH, Serraf I, Sihachakr D (1998) Adventitious shoot regeneration from leaf explants of an African clone of cassava (*Manihot esculenta* Crantz) and analysis of the conformity of regenerated plants. Plant Cell Tiss Org 53:205–211. doi:10.1023/A:1006083514209

[cit0012] Raemakers C, Amati M, Staritsky G, Jacobsen E, Visser R (1993) Cyclic somatic embryogenesis and plant regeneration in cassava. Ann Bot. doi:10.1006/anbo.1993.1037

[cit0013] Taylor N, Gaitan-Solis E, Moll T, Trauterman B, Jones T, Pranjal A, Trembley C, Abernathy V, Corbin D, Fauquet CM (2012) A Highthroughput platform for the production and analysis of transgenic cassava (*Manihot esculenta*) plants. Trop Plant Biol 5:127–139

[cit0014] Tilquin JP (1979) Plant regeneration from stem callus of cassava. Can J Bot 57:1761–1763. doi:10.1139/b79-216

[cit0015] Xu L, Huang H (2014) Genetic and epigenetic controls of plant regeneration. Curr Top Dev Biol 108:1–33. doi:10.1016/B978-0-12-391498-9.00009-7 24512704

